# Surface pseudo-3D time-lapse ERT reveals the spatiotemporal evolution mechanism of lagged water inrush in ultra-thick coal seams

**DOI:** 10.1038/s41598-026-53069-3

**Published:** 2026-05-16

**Authors:** Yuteng Li, Jianyuan Cheng, Yunhong Wang, Jingjin Lu, Zhengfei Wu, Jiajia Zhao, Zhe Fang, Yao Liu, Ruijing Dong

**Affiliations:** 1 China Coal Research Institute, Beijing, 100013 China; 2 CCTEG Xi’an Research Institute (Group) Co., Ltd., Xi’an, 710077 China; 3 Jilin University, Changchun, 130061 China

**Keywords:** Ultra-thick coal seam, Lagged water inrush, Time-lapse ERT, Pseudo-3D imaging, Funnel-shaped channel, Aeolian sand area, Energy science and technology, Engineering, Natural hazards, Solid Earth sciences

## Abstract

The development of water-conducting fractures induced by fully mechanized top-coal caving mining of ultra-thick coal seams in the aeolian sand area of northern Shaanxi is characterized by significant nonlinearity and hysteresis, making water inrush disasters highly concealed and difficult to predict. To overcome the challenges of high-resistance shielding from dry surface sand and the extraction of weak deep signals, this study established a surface pseudo-3D time-lapse high-density electrical resistivity tomography (ERT) monitoring system at the 122105 working face of Caojiatan Coal Mine, utilizing the “artificial wet soil + deep-buried electrode” technique. Combined with anisotropy-constrained inversion and time-lapse ratio imaging technology, the dynamic process of mining-induced overburden failure was visualized and verified by in-situ mine hydrological data. The results indicate that: (1) The center of water inrush is not located at the mining advance line but exhibits a significant “spatiotemporal lag” characteristic, with a lag distance of approximately 110 m, which aligns closely with the limit breaking span of the high-position key stratum. (2) The lagged water inrush channel presents a “funnel-shaped” structure (large at the top and small at the bottom) in 3D space, revealing that its formation results from the fluid-solid coupling connection between the upper separation “catchment basin” and the lower shear “diversion pipe” at the moment of key stratum breaking. (3) The moment of channel connection captured by resistivity imaging (T2) is perfectly synchronized with the onset of the mine water inflow surge, and the water inflow rapidly reached its peak (310 m³/h) within the subsequent 24–48 h, verifying the significant consistency between the flow field and the geoelectric field response. (4) The water-conducting channel in the goaf possesses self-healing properties; as the lag distance exceeds 150 m, the bottom of the channel closes preferentially due to compaction of the caving zone. Based on these findings, a prevention concept of shifting the monitoring field of view to 0–150 m behind the working face is proposed, along with a stereoscopic control strategy of “high-level interception in the funnel zone and low-level drainage in the compacted zone.”

## Introduction

With the continuous westward shift of China’s energy development strategy, the Jurassic coalfield in Northern Shaanxi has been established as a core strategic base for national coal capacity release. The geological environment in this region possesses significant unique characteristics: the surface is widely covered by thick Quaternary aeolian sand, resulting in an extremely fragile ecological environment; meanwhile, the underground coal seams exhibit typical characteristics of “shallow burial depth, thin bedrock, and extra-thick coal seams”^1,2^. To guarantee production capacity and resource recovery rates, large mining height or fully mechanized top-coal caving mining methods are widely employed in this region for high-intensity mining. Particularly for ultra-thick coal seams exceeding 10 m, the large space created by single-pass mining leads to violent and complex overburden movement, which easily induces a nonlinear and super-normal “explosive” development of the roof water-conducting fracture zone^3^. Once the fracture zone reaches the overlying Quaternary Salawusu Formation aquifer, it will not only cause the loss of groundwater resources and aggravate ecological deterioration but also likely trigger serious mine water inrush accidents. Therefore, in the ecologically fragile western area, accurately identifying the dynamic development height, spatial morphology, and evolution laws of the water-conducting fracture zone in ultra-thick coal seam mining is a key prerequisite for realizing “water-preserved coal mining” and intrinsic mine safety^4,5^.

Currently, regarding the detection of development characteristics of water-conducting fracture zones, engineering practice still mainly relies on traditional means such as underground upward borehole water injection leakage observation, borehole TV imaging, and drilling fluid consumption observation. Although these methods possess high accuracy at vertical fixed points, they are essentially static “single-point” observations with a limited field of view. They involve large engineering volumes and high costs, and it is difficult to capture the full process of dynamic evolution and the spatial 3D morphology of the fracture field as the working face advances^6–9^. In contrast, High-density Electrical Resistivity Tomography (ERT), by virtue of its high sensitivity to electrical differences such as rock porosity, water saturation, and fracture development degree, has become an effective geophysical tool for obtaining spatial information on roof failure^10–12^.

However, in the typical aeolian sand coverage area of Northern Shaanxi, conducting surface electrical monitoring faces the dual challenges of “complex geological mechanisms” and a “harsh detection environment.” First is the particularity of the overburden failure mechanism in ultra-thick coal seams. Traditional empirical formulas are mostly established based on the slice mining of medium-thick coal seams. However, for ultra-thick coal seams with a single mining thickness exceeding 10 m, the release of huge free space may cause synchronous composite breakage of the low-level immediate roof and the mid-level key stratum. Consequently, the fracture development height often breaks through the linear prediction range, presenting a nonlinear “jumping” growth characteristic^13–15^. Second is the strong shielding effect of the surface high-resistance layer. The huge thick and dry aeolian sand layer results in extremely high grounding resistance, making it difficult for the supply current to be effectively injected underground. This causes the effective signal of the target layer below a burial depth of 200 m to be extremely weak with an extremely low signal-to-noise ratio. Furthermore, surface electrical inhomogeneity easily forms false anomalies in the deep sections, making it difficult for conventional static inversion to effectively distinguish geological background noise from real, weak mining-induced anomalies^16–18^.

To address the aforementioned problems, Time-lapse Electrical Resistivity Tomography (Time-lapse ERT) provides a new approach for the extraction of deep weak anomalies. This method does not rely on the absolute resistivity value at a single moment but utilizes the resistivity ratio between the post-mining and pre-mining stages for imaging through repeated observations of the same survey line. This “relative change” imaging strategy can effectively eliminate background geological noise and static distortion caused by surface aeolian sand, significantly amplifying the dynamic electrical response caused by mining failure (fracture opening or water filling)^19,20^. In addition, given that the 2D section of a single survey line is insufficient to reflect the overall failure morphology of a wide fully mechanized top-coal caving working face, constructing a pseudo-3D observation system by deploying multiple parallel survey lines, combined with anisotropy-constrained inversion technology, can facilitate stereoscopic control of the roof failure range.

In view of this, this paper takes the 122105 fully mechanized top-coal caving working face of the Caojiatan Coal Mine in Northern Shaanxi as the engineering background to conduct research on the technical bottlenecks of deep detection in aeolian sand areas and the scientific problems regarding the water inrush mechanism in ultra-thick coal seams. The burial depth of the coal seam in this working face is approximately 300 m, and the single-pass mining thickness reaches 11.8 m. This paper proposes and adopts the “deep-buried electrode” technique to construct a surface pseudo-3D high-density electrical observation system to overcome the high-resistance shielding problem. Based on long-time sequence monitoring data from four key nodes—“background period,” “fracture outbreak period,” “water filling period,” and “post-mining lagged evolution period”—and utilizing a 3D time-lapse inversion algorithm, this paper focuses on analyzing the vertical evolution and planar distribution characteristics of roof apparent resistivity during the mining process. The study aims to quantitatively reveal the nonlinear development laws of water-conducting fracture zones and the lagged water inrush mechanism under the conditions of ultra-thick coal seam mining, construct a time-varying electrical response model of “high-resistance breaking — low-resistance water filling — self-healing closure” of the mining overburden, and provide a reliable geophysical basis for the identification and prevention of water hazards under similar geological conditions.

## Geological background and methodology

### Mine geology and hydrogeological conditions

#### Stratigraphic structure and electrical characteristics

**Fig. 1:**
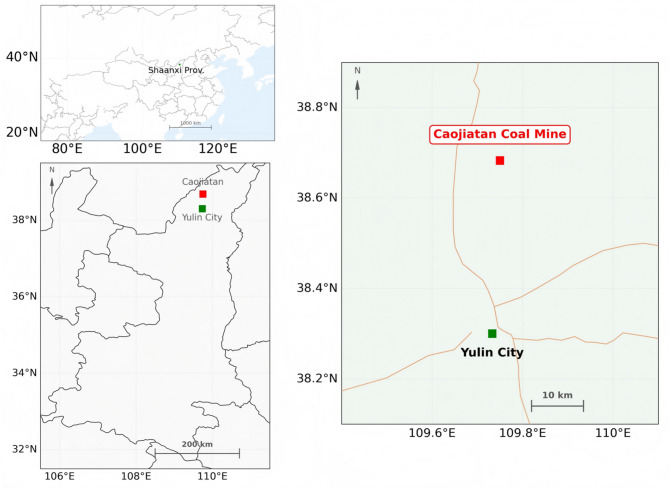
Traffic location and study area map of Caojiatan Coal Mine (The maps in this figure were generated using **Python** (Version 3.9, https://www.python.org/) and the **Cartopy** library. Geographic data (boundaries, rivers, and roads) were sourced from the **Natural Earth** dataset (https://www.naturalearthdata.com/), which is in the public domain.).

**Fig. 2:**
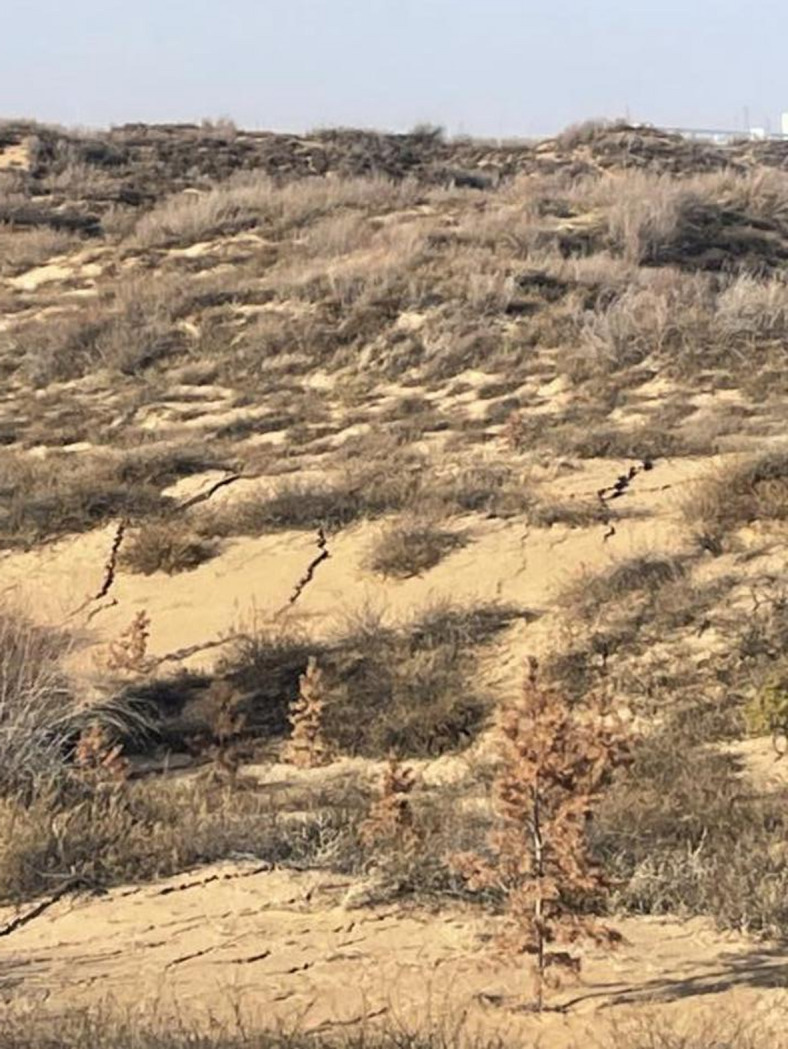
Typical aeolian sand landform in the study area.

The study area is situated at the Caojiatan Coal Mine in Yulin City, Shaanxi Province (Fig. 1), located on the southern margin of the Mu Us Desert. The surface is extensively covered by extremely thick Quaternary aeolian sand $\:{(\mathrm{Q}}_{4}^{\mathrm{e}\mathrm{o}\mathrm{l}})$(Fig. 2). Due to the arid climate with scant rainfall, the surface sandy soil is extremely dry and loose, resulting in exceptionally poor electrical conductivity. The grounding resistance typically reaches thousands of ohms, constituting a natural “high-resistance shielding layer” for surface electrical exploration, which severely hinders the effective injection of supply current into the deep underground.

According to borehole exposure data, the primary mining target of the 122105 working face is the No. 2^− 2^ coal seam of the Jurassic Yan’an Formation. The overlying strata, ordered from oldest to newest (code S6–S1), are as follows:

Zhiluo Formation ($\:S6$): Composed mainly of interbedded gray-green medium-coarse sandstone and silty mudstone, serving as the immediate roof and main roof of the coal seam.

Anding Formation ($\:S5$): The lithology is dominated by purple-red mudstone and sandy mudstone. The rock mass is dense and intact, acting as a key regional aquifuge that plays a crucial role in blocking the downward seepage of upper water bodies.

Neogene Baode Formation ($\:S3$) and Quaternary Lishi Formation ($\:S2$): Located above the Anding Formation, the lithology consists mainly of light red to brownish-red clay (red soil) and light brownish-yellow sub-sandy soil (loess), functioning as a relative aquifuge or weak aquifer.

Quaternary Salawusu Formation ($\:S1$) and Aeolian Sand: The Salawusu Formation mainly consists of gray-yellow fine-silty sand with strong water abundance; the surface is covered by aeolian sand.

The Quaternary Salawusu loose aquifer and its underlying weathered bedrock zone (the weathered fracture development zone at the top of the Anding or Zhiluo Formations, with a burial depth of approximately 50–150 m) are the protected aquifer horizons of primary concern in this study.

**Fig. 3:**
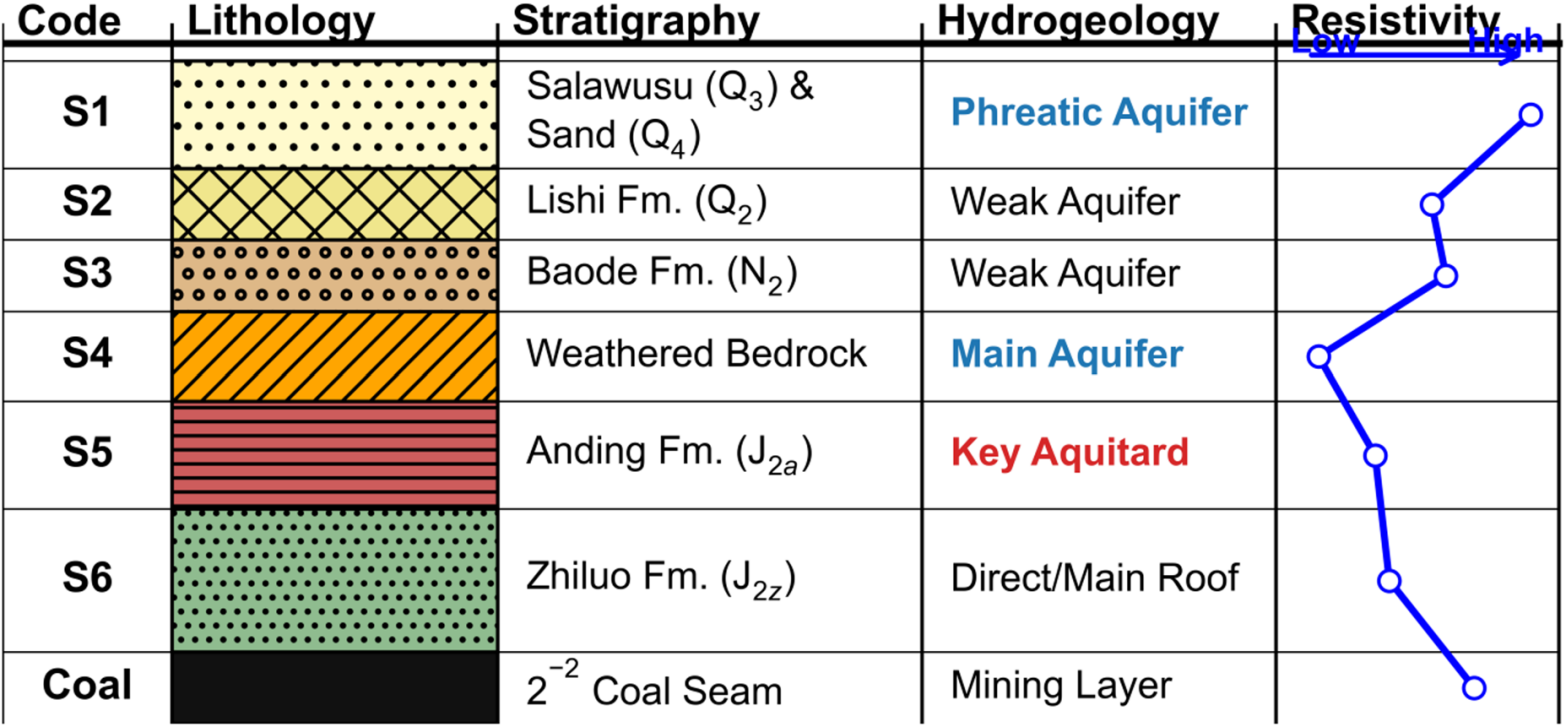
Schematic diagram of the distribution of main overlying strata of the 122105 working face.

The electrical properties of the strata exhibit a clear layered distribution characteristic: the dry surface aeolian sand manifests as extremely high resistivity ($\:\rho\:>1000{\Omega\:}\cdot\:m$); the middle-shallow Salawusu Formation and water-rich weathered bedrock layer manifest as relatively low resistivity; and the deep Zhiluo and Anding Formation bedrock manifests as medium-high resistivity (Fig. 3). This significant “High-Low-Medium” electrical difference provides a favorable geophysical premise for utilizing apparent resistivity variations to identify the structural evolution of aquifers subject to mining-induced failure.

#### Characteristics of fully mechanized top-coal caving in ultra-thick coal seams

The 122105 working face has a strike length of 5965 m and a dip length (width) of 300 m. The average thickness of the coal seam is 11.8 m, with a burial depth of 280–320 m and a dip angle of less than 1°, classifying it as a near-horizontal ultra-thick coal seam. The working face adopts the large mining height fully mechanized top-coal caving technology, with a cutting height of 5.8 m and a caving height of 6.0 m, resulting in a single-pass extraction thickness of up to 11.8 m. Such a massive release of mining space in a single pass causes the overlying strata to lose the buffering conditions for progressive settlement, which easily induces composite breakage and overall cutting of key strata.

Theoretical predictions and experience from adjacent mines indicate that the development height of the water-conducting fracture zone in such ultra-thick coal seam mining will far exceed the values predicted by empirical formulas. It is highly likely to propagate upward to reach the weathered bedrock or even the bottom boundary of the Salawusu Formation. Therefore, the core objective of this monitoring is to identify whether the top boundary of the fracture zone breaches the Anding Formation aquifuge and to accurately capture its hydraulic connection with the upper aquifers (within a depth range of 250 m).

### Ground Pseudo-3D monitoring system design

To address the detection conflict between the “large burial depth of the coal seam” and the “strong shielding of surface aeolian sand” in the study area, this project abandoned the traditional single-line 2D detection mode. Instead, a surface pseudo-3D observation system based on a long-line array was designed, and a targeted deep-buried electrode technique was implemented to ensure the high-quality acquisition of weak deep signals.

#### Observation system parameters and layout

**Fig. 4:**
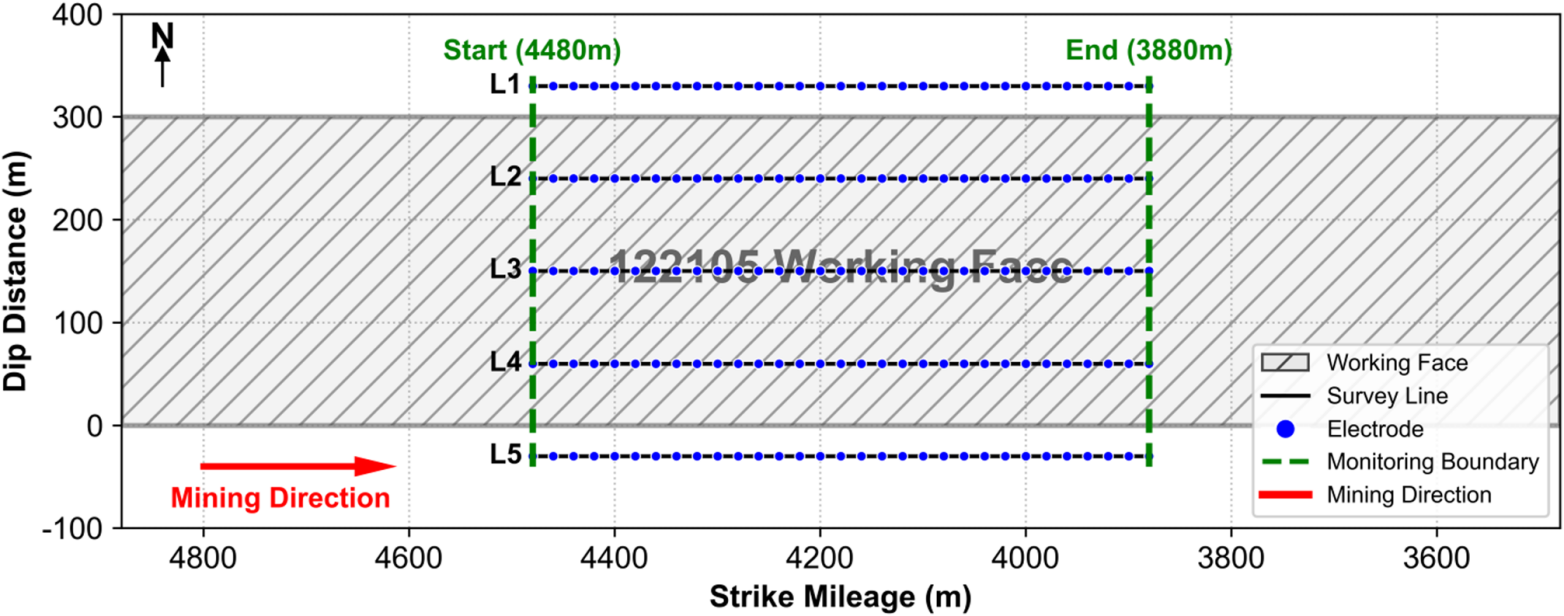
Plan view of surface pseudo-3D electrical survey lines relative to the working face.

To achieve stereoscopic control over the mining-induced failure range of the 122105 working face, five high-density electrical survey lines (labeled L1–L5) were deployed parallel to the strike of the working face to construct a pseudo-3D observation network (Fig. 4):

Survey line distribution: Line L3 was arranged above the dip centerline of the working face as the main monitoring axis; Lines L1 and L5 were arranged above the haulage gate and return air gate, respectively, to control the failure characteristics of the two side boundaries of the goaf; Lines L2 and L4 were located in the intermediate regions.

Survey line scale: The length of a single survey line was set to 1200 m. This length design not only completely covered the core mining area but also included the advanced abutment pressure zone ahead of the working face and the stable zone behind the goaf, meeting the data truncation requirements for long-distance time-lapse monitoring.

Spatiotemporal resolution: To balance vertical layering accuracy and detection depth, the electrode spacing was set to 10 m. To control the failure range in the dip direction of the working face, the line spacing was set to 90 m, with a total control width of 360 m, effectively covering the main influence domain in the dip direction.

Array and depth: The Wenner-Schlumberger array, characterized by strong anti-interference ability and high vertical resolution, was selected for data acquisition. The maximum separation factor ($\:n$) was taken up to more than 30 levels, and the effective Depth of Investigation (DOI) for inversion was approximately 250 m. Although this depth did not directly expose the coal seam floor at 300 m, its observational field of view completely covered the top horizon of the upward development of the water-conducting fracture zone (estimated height > 200 m) as well as key aquifer regions such as the Salawusu Formation and weathered bedrock. Therefore, this observation system fully meets the requirements of the core research objective: “monitoring whether the fracture zone affects the aquifers.”

#### “Deep-Buried Electrode” technique targeting aeolian sand areas

**Fig. 5:**
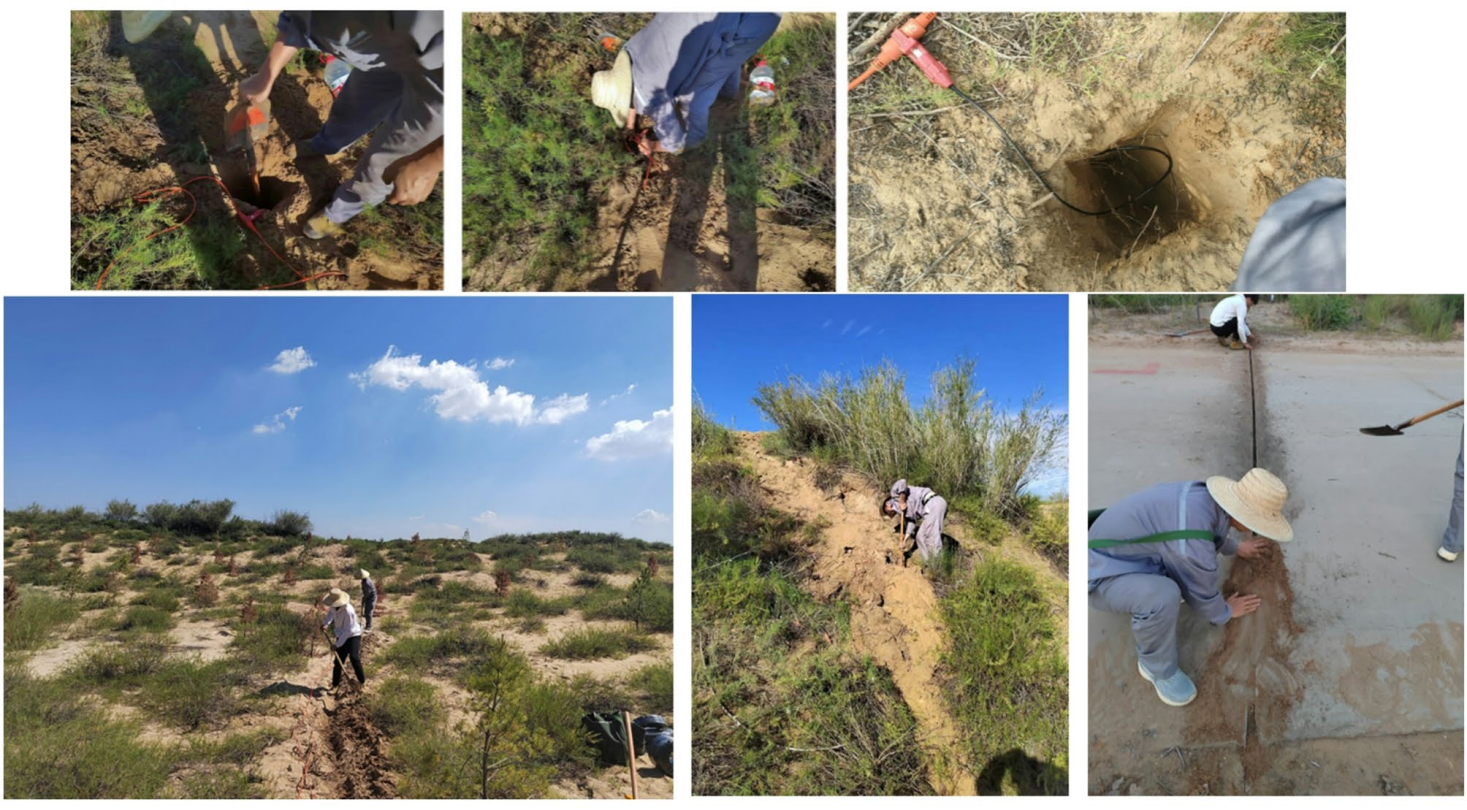
Field photos of deep-buried electrode installation in aeolian sand area.

The dry aeolian sand covering the surface of the study area serves as a critical bottleneck restricting the quality of electrical data. Field tests indicated that when employing the conventional surface direct insertion method, the contact resistance between the electrode and the sand generally exceeded $\:2000{\Omega\:}$. This made it difficult to inject supply current into the ground (typically $\:<10mA$), causing useful deep signals to be easily submerged by background noise, resulting in an extremely low signal-to-noise ratio (SNR). To overcome this physical challenge, this project adopted a deep-buried electrode placement technique featuring “manual excavation + wet soil coupling,” as shown in Fig. 5.

Positioning and Excavation: The electrode burial depth (0.6–1.0 m) was determined based on the vertical moisture stratification of the surface aeolian sand. Field observations during installation revealed that the uppermost 0.5–0.8 m consists of extremely dry yellow sand with negligible moisture content and high resistivity ($\:>1{0}^{4}\:{\Omega\:}$). Beneath this desiccated layer, the undisturbed wet silt/soil layer was exposed. By manually excavating through the dry sand and implanting the copper electrodes directly into the underlying wet soil, combined with 3% saline coupling, the grounding resistance was successfully reduced from thousands of ohms to less than 500 $\:{\Omega\:}$. This ensured the stable injection of the supply current (200–500 mA) required for deep-seated signal extraction.

Implantation and coupling: Copper electrodes were driven vertically into the wet soil at the bottom of the pit to ensure tight contact between the electrode and the undisturbed soil.

Backfilling and compaction: The electrode pits were backfilled with wet soil and compacted in layers. When necessary, saltwater was poured around the electrodes to further reduce the grounding resistance.

Following the implementation of this technique, the average grounding resistance of electrodes across the entire area decreased significantly to below $\:500{\Omega\:}$, and the supply current increased to the level of $\:200-500\:\mathrm{m}\mathrm{A}.$ This measure fundamentally resolved the high-resistance shielding issue and significantly improved the SNR of the raw data, laying a solid data foundation for the subsequent extraction of weak mining-induced electrical responses at a depth of 250 m.

To quantitatively highlight the advantages and innovative value of this technique, a comparative analysis of the actual field test effects between the conventional electrode layout and the proposed method is summarized in Table 1. As shown, the traditional surface insertion method is practically inadequate for deep exploration in aeolian sand areas due to its high contact resistance and severely limited supply current (< 10 mA). In contrast, the proposed “artificial wet soil + deep-buried electrode” technique fundamentally eliminates the high-resistance shielding effect. By increasing the supply current by more than an order of magnitude, this technique guarantees the reliability of the deep geoelectric data inversion.

**Table 1:** Comparison of technical parameters and field performance between the conventional surface insertion and the proposed method.

Parameters	Conventional surface insertion method	Proposed method (Deep-buried + wet soil coupling)
Coupling medium	Dry aeolian sand	3% saline solution + wet silt/soil
Electrode burial depth	0.1–0.2 m (Surface layer)	0.6–1.0 m (Penetrating the dry sand layer)
Grounding resistance	> 2000$\:{\Omega\:}$ (often > 10^4^$\:{\Omega\:}$)	< 500$\:{\Omega\:}$
Average supply current	< 10 mA	200–500 mA
Data quality & SNR	Extremely low (deep signals submerged by noise)	High SNR (meets the requirement for 250 m exploration)

### 3D time-lapse inversion and imaging strategy

To accurately extract the weak deep dynamic response induced by mining from the raw apparent resistivity data, this study formulated a three-stage data processing strategy: “core area truncation — anisotropy-constrained inversion — time-lapse ratio imaging.”

#### Core area data reconstruction and establishment of relative coordinates

**Fig. 6:**
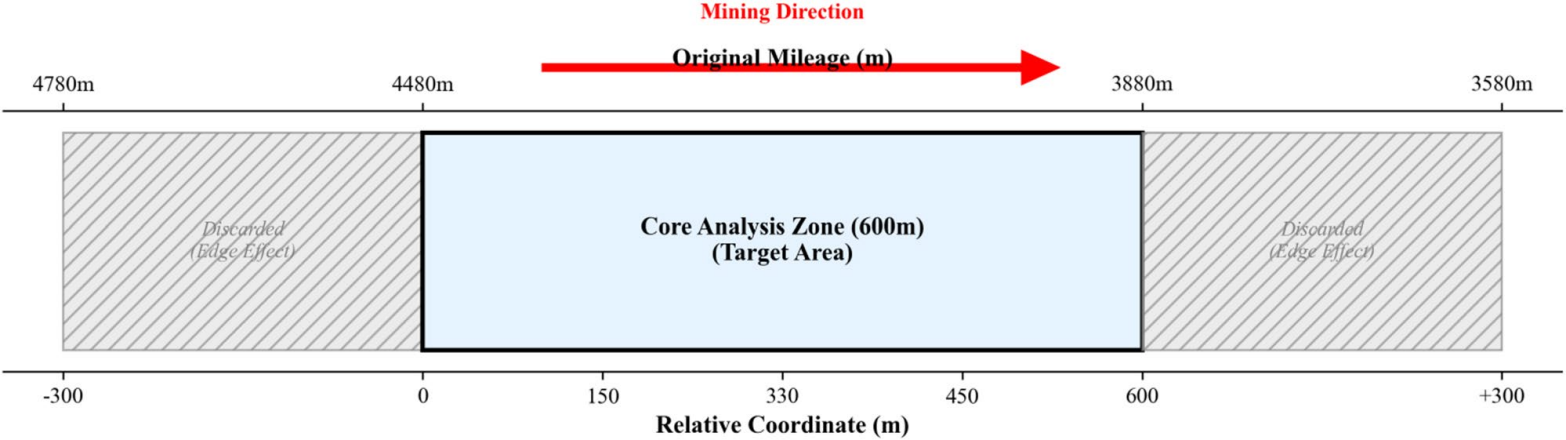
Schematic diagram of data truncation strategy and relative coordinate system mapping.

In long-distance electrical inversion, data at both ends of the survey line are often limited by insufficient coverage, leading to significant uncertainty at the edges of the inversion model. To ensure the accuracy of subsequent analysis, this project adopted a strategy of “long-line observation and core area truncation” (Fig. 6):

Data Truncation: Data from unstable sections at both ends of each survey line were excluded. Only the middle 600 m section (corresponding to original mileage 4480 m–3880 m), which possessed the most stable data quality and was most significantly affected by mining, was retained as the core inversion domain.

Establishment of Relative Coordinate System: To facilitate a unified description of the spatial relationship between the working face advancement position and fracture evolution, a relative coordinate system was established. The original mileage 4480 m was defined as the relative coordinate origin (0 m), and the original mileage 3880 m was defined as the endpoint (600 m). In this coordinate system, the working face advances along $\:0\,m\to\:600\,m$ direction.

#### Anisotropy-constrained 3D inversion algorithm

The core principle of the inversion is to seek the minimum value of the model objective function ${\mathrm{\boldsymbol{\Phi}}}\left( {\mathbf{m}} \right)$. This objective function comprises two parts: the data misfit term and the model roughness constraint term. Its mathematical expression is given by:

$${\mathrm{\boldsymbol{\Phi}}}\left( {\mathbf{m}} \right)={\left\| {{{\mathbf{W}}_d}\left( {{{\mathbf{d}}_{obs}} - {\mathbf{f}}\left( {\mathbf{m}} \right)} \right)} \right\|^2}+\lambda {\left\| {{{\mathbf{C}}_m}{\mathbf{m}}} \right\|^2}$$

Where:

${{\mathrm{d}}_{{\mathrm{obs}}}}$ is the observed apparent resistivity data vector; ${\mathbf{f}}\left( {\mathbf{m}} \right)$ is the forward response of the current geoelectric model ${\mathbf{m}}$; ${{\mathbf{W}}_d}$ is the data weighting matrix (typically based on the reciprocal of data errors); $\lambda$ is the damping factor, used to balance the data misfit and model smoothness; ${{\mathbf{C}}_m}$ is the smoothing constraint matrix, which is key to resolving the issue of wide line spacing.

In isotropic inversion, the smoothing weights of the model in the $x,y$ and *z* directions are equal. However, in this study, to suppress abrupt variations between sparse survey lines, we introduced directional weighting coefficients (${\alpha _x},{\alpha _y},{\alpha _z}$) into ${{\mathbf{C}}_m}$ and set ${\alpha _y}>{\alpha _x} \approx {\alpha _z}$ (where *y* represents the dip direction of the working face, perpendicular to the survey lines). By enhancing the model smoothing constraint in the dip direction, the data gaps caused by the 90 m line spacing were effectively “bridged,” thereby constructing a pseudo-3D resistivity data volume that is geologically reasonable and spatially continuous.

$$R\left( {x,y,z} \right)=\frac{{{\rho _{ti}}\left( {x,y,z} \right) - {\rho _{t0}}\left( {x,y,z} \right)}}{{{\rho _{t0}}\left( {x,y,z} \right)}} \times 100\%$$

To address the spatial sparsity between the five parallel survey lines (90 m spacing), the directional smoothing weights in the anisotropy-constrained inversion were set as ${w_x}=1.0$, ${w_y}=3.0$, and ${w_z}=1.0$. The higher weight assigned to the dip direction (*y*) helps to stabilize the 3D model by suppressing abrupt inter-line fluctuations, ensuring a geologically continuous reconstruction of the water-conducting channels across the entire monitoring area.

#### Time-lapse resistivity ratio imaging technology

The inhomogeneity of surface aeolian sand generates stationary static high- or low-resistivity background anomalies on the inversion sections. These static backgrounds often obscure the weak mining-induced signals at depth.

To achieve the goal of “eliminating background interference and highlighting dynamic changes,” this paper adopts the time-lapse ratio imaging technique. The inversion result from August 20 (when the working face had advanced 150 m into the monitoring range) was selected as the background baseline model (${\rho _{t0}}$). The relative resistivity change rate (*R*) for subsequent monitoring times (${t_i}$) was calculated as follows:

$$R\left( {x,y,z} \right)=\frac{{{\rho _{ti}}\left( {x,y,z} \right) - {\rho _{t0}}\left( {x,y,z} \right)}}{{{\rho _{t0}}\left( {x,y,z} \right)}} \times 100\%$$

Where *R* represents the resistivity change rate. This parameter possesses distinct physical significance:

$R>0$ (Positive anomaly): Indicates the occurrence of bed separation or fracturing in the rock strata, where conductive channels are filled with air, leading to an increase in resistivity. A larger *R* value suggests a greater fracture aperture.

$R<0$ (Negative anomaly): Indicates that fractures are filled with low-resistivity media (e.g., mine water or mud), or that pores in the goaf have closed due to strata compaction.

Compared with conventional static inversion, the time-lapse ratio imaging strategy significantly enhances the reliability of deep signal extraction. By utilizing the pre-mining state (${t_0}$) as a baseline, static inversion artifacts and distortions caused by surface high-resistance aeolian sand are mathematically canceled. This ensures that the dynamic anomalies captured at a depth of 250 m are physically rooted in the mining-induced overburden failure rather than computational noise.

## Spatiotemporal evolution characteristics and analysis of overburden resistivity under mining

To intuitively demonstrate the spatiotemporal evolution characteristics of the mining-induced water-conducting fracture zone, this paper performs an in-depth analysis by extracting vertical slices at different monitoring times and horizontal slices at different horizons based on the constructed pseudo-3D time-lapse resistivity data volume.

### Vertical development and evolution characteristics of the water-conducting fracture zone

**Fig. 7:**
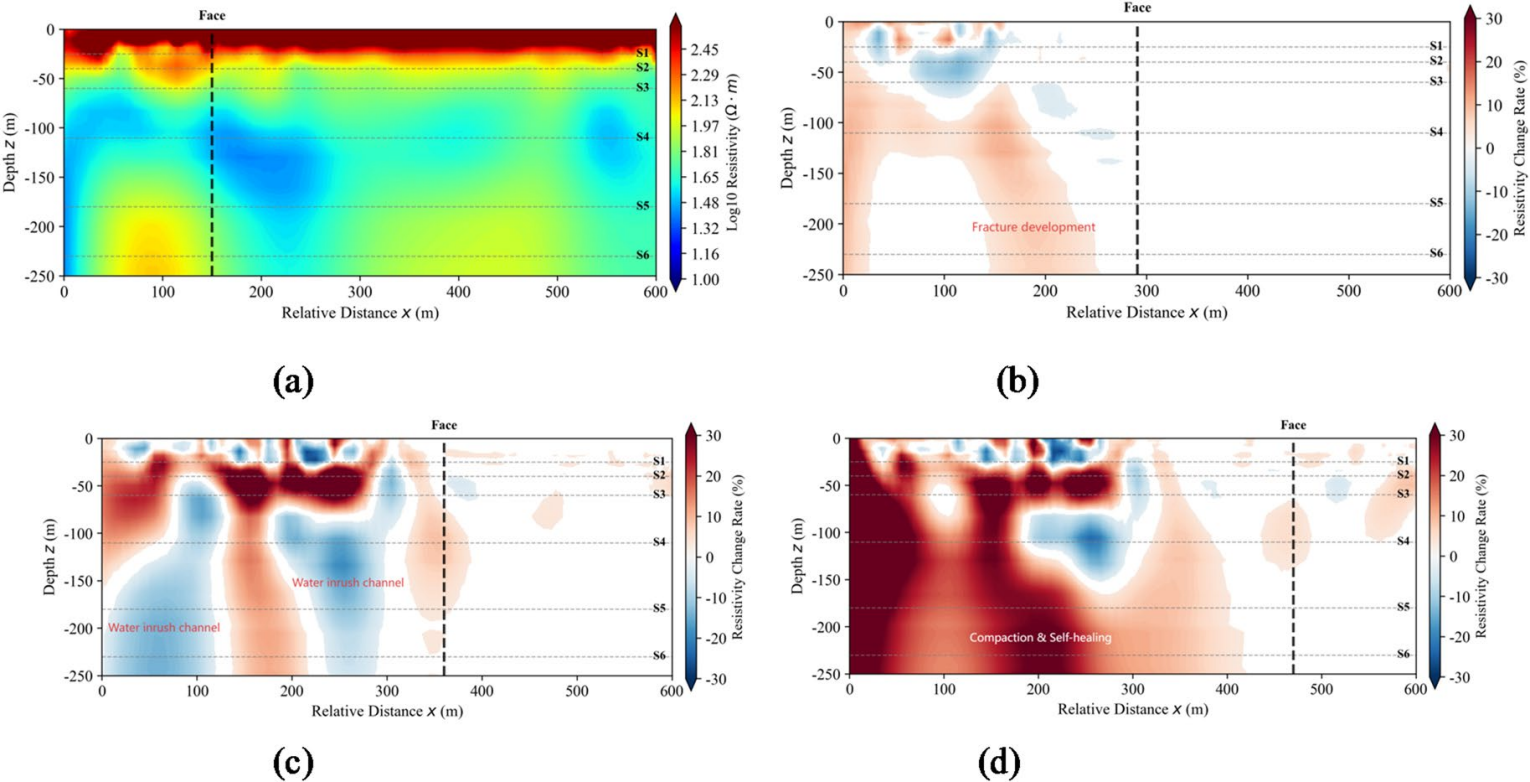
Vertical evolution slices of resistivity and change rate along Line L3 at different mining stages. (a) T0: Background apparent resistivity, (Aug. 20), (b) T1: Resistivity change rate during fracture development, (Sep. 4), (c) T2: Resistivity change rate during water inrush breakthrough, (Sep. 12), (d) T3: Resistivity change rate during post-mining stabilization, (Sep. 25)

The vertical slice corresponding to the position of Survey Line L3, located at the dip centerline of the working face, was extracted from the 3D inversion volume to analyze the geoelectric field response characteristics at four key mining stages. Figure 7 illustrates the spatiotemporal evolution process of the relative resistivity change rate ($\:R$) with the advancement of the working face.

#### Characterization of background geoelectric model and stratigraphic division (Stage T0)

Figure 7(a) presents the resistivity inversion imaging result at T0 (Aug. 20) when the working face had advanced to $x=150{\mathrm{m}}$. Serving as the baseline state for subsequent dynamic monitoring, this section is overlaid with horizontal stratigraphic division lines (S1–S6), clearly delineating the “High-Low-Medium” three-zone electrical stratigraphic structure of the overburden in the study area:

(1) Shallow high-resistivity overburden (Surface to S3, z= $0\sim - 70{\mathrm{~m}}$)

This horizon exhibits significant high-resistivity characteristics as a whole. Combined with the borehole column data of the mining area, S1 (Salawusu Formation), S2 (Lishi Formation), and S3 (Baode Formation) are primarily composed of Quaternary aeolian sand, loose sediments, and loess/red clay. Since these strata are located above the water table, their moisture content is extremely low, and the pore media are mainly filled with air, resulting in typical high insulation characteristics in the background field.

(2) Middle low-resistivity shielding layer (S3–S5, z= $- 70\sim - 110{\mathrm{~m}}$)

Between S3 and S5 (near z ≈ $- 110{\mathrm{~m}}$), a continuous and stable relatively low-resistivity band is developed. Geological data confirm that this horizon corresponds to the S4 weathered bedrock section, which serves as the primary aquifer carrier in the mining area. Influenced by the dual effects of developed weathering fissures and high salinity (mineralization) of pore water, the conductivity of this horizon is significantly enhanced. Vertically, it constitutes a distinct low-resistivity electrical shielding layer and acts as the key water-bearing horizon for subsequent water inrush disasters.

(3) Deep bedrock layer (S5–S6, $z< - 110{\mathrm{m}}$)

The deep strata are mainly composed of S5 (Anding Formation) and S6 (Zhiluo Formation). The lithology is dominated by interbedded dense sandstone and mudstone, generally presenting a medium-high resistivity background. Notably, a local relative low-resistivity anomaly zone exists within the S6 Zhiluo Formation (x = $160\sim 280{\mathrm{~m}}$, z < $- 150{\mathrm{~m}}$). Considering that this region is located deep ahead of the working face and has not yet been significantly damaged by advanced abutment pressure, it is inferred that this anomaly is attributed to the lateral lithological heterogeneity within the sedimentary strata. Specifically, it corresponds to muddy sandstone lenses or sandstone bands with developed primary pore structures within the Zhiluo Formation, resulting in apparent resistivity locally lower than that of the dense surrounding rock.

#### Characteristics of explosive development of mining-induced fractures (Stage T1)

Figure 7(b) presents the resistivity change rate imaging result at T1 (Sep. 4) when the working face had advanced to $x=291{\mathrm{m}}$. At this moment, mining disturbances reached an active peak period. Compared with the T0 background field, the geoelectric response exhibits significant “positive high-resistivity evolution” characteristics (in the figure, weak fluctuations within the change rate range of $- 5\% \sim 5\%$ are filtered out as a white background):

(1) High-resistivity fracturing response of overburden above the goaf

In the overburden above the goaf behind the working face ($x=0\sim 291{\mathrm{~m}}$), an extensive zone of resistivity increase anomalies manifests (red areas, where the change rate is generally $>10\%$). Based on the mechanics of mining rock, with the high-intensity extraction of the ultra-thick coal seam, the immediate roof and main roof undergo periodic caving. This induces strong tensile and shear failure in the overlying strata, generating abundant bed separations and vertical breaking fractures. Since massive water inrush has not yet occurred at this stage, the newly generated fracture spaces are primarily filled with high-resistance air, which effectively severs the original conductive paths of the rock strata. Consequently, this manifests as a strong positive high-resistivity anomaly on the imaging section.

(2) Through-layer development characteristics of the water-conducting fracture zone

In terms of vertical distribution morphology, the high-resistivity fracture zone has completely penetrated the S5 Anding Formation. It exhibits a “flame-shaped” pattern, aggressively protruding upward into the interior of the S4 Weathered Bedrock, with the top boundary of the anomaly zone approaching the bottom interface of the S3 Baode Formation (vertical depth of approximately $- 80{\mathrm{~m}}$). This characteristic indicates that the water-conducting fracture zone is undergoing an explosive upward development process and has already impacted key aquifer horizons, posing an imminent risk of connecting with the upper aquifers.

(3) Lateral differences in the advanced influence zone

In contrast, in the unmined solid coal area ahead of the working face ($x>291m$), the amplitude of the resistivity change rate remains relatively small and generally stays within the background threshold. This indicates that although the advanced abutment pressure causes stress concentration, it has not yet induced large-scale plastic failure in the deep strata sufficient to significantly alter the geoelectric structure. This significant lateral difference further confirms the high spatiotemporal correlation between the red high-resistivity anomalies and the processes of mining-induced caving and fracture opening.

#### Breakthrough of water-conducting channels and response characteristics of water inrush disaster (Stage T2)

Figure 7(c) presents the evolution results of the apparent resistivity change rate at T2 (Sep. 12), when the working face had advanced to $x=360{\mathrm{m}}$. At this moment, the monitoring section revealed a fundamental mutation in the electrical characteristics of the mining overburden. A typical “polarity reversal” phenomenon emerged, marking the official transition of the overburden movement state from the “fracture development period” to the “water inrush breakthrough period”:

(1) Polarity reversal of electrical anomalies

Compared with Stage T1, although the original strip-shaped high-resistivity anomaly ($x=150\sim 250{\mathrm{m}}$, dipping to the left) above the goaf remained near the S2 stratum ($x=130\sim 280{\mathrm{m}}$), the most significant change lies in the rapid evolution of strong low-resistivity anomaly zones (blue areas) on both sides of the high-resistivity anomaly. This phenomenon indicates that the fracture space has been filled by ascending water, causing the dominant factor of the medium’s electrical properties to shift from “mining-induced fractures (air)” to “fracture water bodies.”

(2) Vertical breakthrough of low-resistivity water-conducting channels

The blue low-resistivity bands exhibit a distinct vertical strip-like morphology, primarily concentrated in the regions of $x=0\sim 120{\mathrm{~m}}$ and $x=220\sim 300{\mathrm{~m}}$. Spatially, their upper extremities closely abut the S3 Baode Formation (at a vertical depth of approximately $- 70{\mathrm{~m}}$). Extending downward along the mining-induced water-conducting fracture zone, they penetrate the S5 Anding Formation and S6 Zhiluo Formation in sequence from shallow to deep, finally reaching the goaf directly.

(3) Geophysical determination of water hazard sources

The aforementioned breakthrough morphology of the low-resistivity anomalies provides direct geophysical evidence confirming that the main recharge sources for this water inrush are the S4 weathered bedrock phreatic water and the lower weak aquifer of the S3 Baode Formation. The continuity of the low-resistivity bands indicates that the water-conducting fracture channels have completely penetrated the overlying aquifuges (Anding and Zhiluo Formations), establishing a stable hydraulic connection. Consequently, this resulted in the inrush of roof aquifer water into the goaf along the fracture channels.

#### Electrical recovery of post-mining overburden and characteristics of residual water distribution (Stage T3)

Figure 7(d) presents the evolution results of the resistivity change rate at T3 (Sep. 25), when the working face continued to advance to $x=470{\mathrm{m}}$. Compared with the “water inrush breakthrough” in Stage T2, the monitoring profile in this stage exhibits significant features of “high-resistivity rebound and low-resistivity contraction,” marking the entry of overburden movement into the “fracture closure and drainage stabilization period”:

(1) High-resistivity response to mining-induced fracture closure and strata dewatering

In the monitoring profile, the vast majority of the area ($x=0\sim 390{\mathrm{~m}}$) shifts to red high-resistivity anomalies. This indicates that after the mining-induced fractures experienced opening and water filling, they entered the compaction and closure stage with the propagation of stress waves and the discharge of fluids:

Strong compaction/dewatering zone (Dark Red): This zone manifests as deep red high-resistivity anomalies in the area of ${\mathrm{x}}=0\sim 30{\mathrm{~m}}$ and ${\mathrm{x}}=160\sim 230{\mathrm{~m}}$. This reveals that the overburden above the open-off cut and the central goaf, after undergoing initial caving and periodic weighting, has seen its fractures gradually compacted and closed, blocking the conductive channels. Simultaneously, as the water inrush process concluded and the fissure water was drained, the resistivity of the rock matrix re-established dominance.

Transitional recovery zone (Light Red): This zone appears light red in the area of ${\mathrm{x}}=40\sim 150{\mathrm{~m}}$and ${\mathrm{x}}=250\sim 390{\mathrm{~m}}$. It indicates that the fractures in this region are in a semi-closed or semi-filled state. With the decrease in water saturation, the electrical characteristics are in a transition stage, recovering from low resistivity to the background value.

Advanced influence zone (White): Most of the area where $x=470\sim 600{\mathrm{~m}}$ appears white, indicating that this region has not yet been severely disturbed by mining stress, and the electrical properties of the strata remain in the undisturbed rock background state.

(2) Spatiotemporal inheritance of residual water accumulation areas

Despite the overall trend of high-resistivity recovery in the profile, a significant blue low-resistivity anomaly persists in the deep region (${\mathrm{x}}=200\sim 320{\mathrm{~m}},{\mathrm{z}}= - 80\sim - 150{\mathrm{~m}}$). Compared with Fig. 7(c), this low-resistivity anomaly exhibits a high degree of spatiotemporal inheritance in terms of spatial position and morphology, neither dissipating nor undergoing polarity reversal over time. Integrated with geological horizon analysis, this region is located at the top of the S5 Anding Formation and within the core of the goaf caving zone. This indicates that while the upper recharge channels (S3/S4) have been blocked due to fracture closure, a stable “goaf water accumulation basin” has formed in this region. The inrushed water converges here; restricted by the floor morphology or the void structure of the caved rock mass, it is difficult to discharge completely within a short period, thereby forming a concealed hazard zone of old goaf water.

### Spatial morphology and distribution characteristics of mining-induced overburden water hazards

**Fig. 8:**
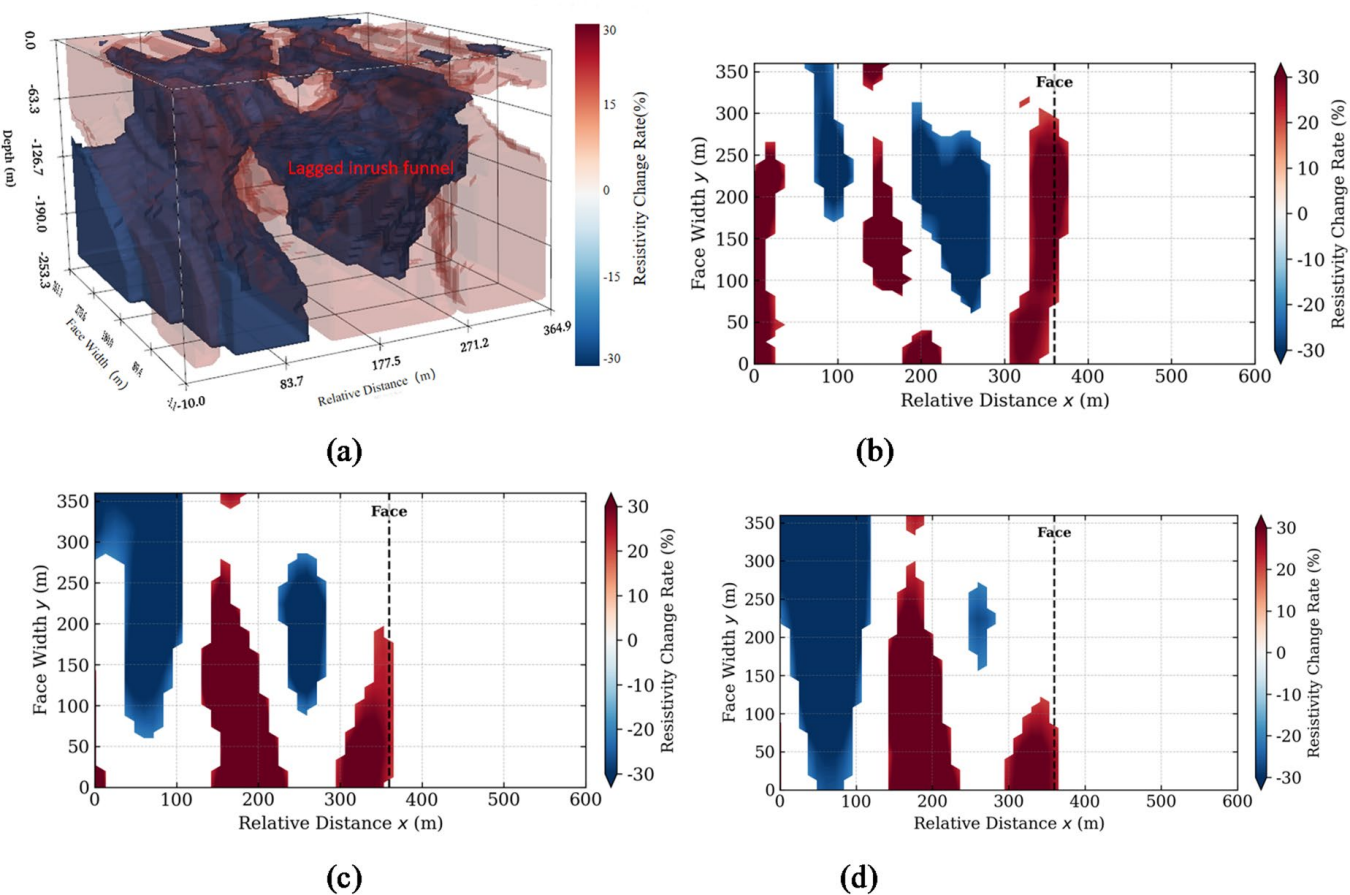
Three-dimensional spatial distribution and planar slice characteristics of apparent resistivity change rate during the water inrush breakthrough period (T2).(The 2D slices were generated using the **Matplotlib** library (https://matplotlib.org/), and the 3D isosurface maps were rendered using the **PyVista** library (https://docs.pyvista.org/) in **Python** (Version 3.9, https://www.python.org/).) (a) 3D isosurfaces of resistivity change rate (Blue bodies: change rate < -5%; Red bodies: change rate > 10%). (b) Horizontal slice at z = -120 m. (c) Horizontal slice at z = -160 m. (d) Horizontal slice at z = -230 m.

Conventional 2D resistivity profiles (as described in Sect.  3.1) fail to fully reveal the lateral heterogeneity and stereoscopic morphology of fluid distribution within the goaf. To intuitively reconstruct the water inrush disaster process, this section reconstructs the overburden apparent resistivity change rate data volume at time T2 using 3D visualization technology, based on time-lapse resistivity monitoring data (Fig. 8).

#### 3D morphological reconstruction of fluid distribution in Goaf and lagged water inrush channels (Stage T2)

As shown in Fig. 8(a), The 3D isosurfaces in Fig. 8(a) were rendered using a filtering threshold of $- 5\%$ for the resistivity change rate. This selection is scientifically grounded in the systematic noise floor of the long-line monitoring system. Data repeatability tests conducted during the background phase (${T_0}$) indicated that instrument stacking errors and environmental electromagnetic interference resulted in a baseline fluctuation of approximately $3\% --5\%$. By masking changes within this $- 5\%$ range, minor measurement artifacts were suppressed, ensuring that the visualized funnel-shaped and water-accumulation bodies represent robust geoelectric responses (where changes typically exceed 10%) rather than computational noise. In Fig. 8(a), the 3D resistivity imaging results at T2 (the water inrush outbreak period) clearly reveal a significant “zonal differentiation” in the electrical structure of the goaf overburden. Within the monitoring range, the fluid distribution in the goaf exhibits a spatial characteristic of the coexistence of “central high-position breakthrough water inrush” and “rear low-position compaction water accumulation.” To decipher the essential difference between these two types of anomalies, a stereoscopic dissection of the internal structure of the goaf was conducted by combining horizontal slices (Figs. 8b–d) of three key horizons: $z= - 120{\mathrm{m}}$ (Top boundary of Anding Formation), $z= - 160{\mathrm{m}}$ (Bottom boundary of Anding Formation), and $z= - 230{\mathrm{m}}$ (Middle of Zhiluo Formation).

1. “Funnel-shaped” Breakthrough Morphology of the Central Lagged Water Inrush Channel ($x \approx 250{\mathrm{m}}$)

Near the relative coordinate $x=250{\mathrm{m}}$ (i.e., lagging behind the working face by approximately 110 m), the core disaster-causing structure of this water inrush event was monitored. The 3D and slice data show that this low-resistivity anomaly exhibits a typical “funnel-shaped” morphology characterized by a “large top and small bottom,” clearly delineating the process of water converging from a high position and discharging intensely to a low position:

Top Catchment Basin ($z= - 120{\mathrm{m}}$, Fig. 8b): At the top boundary of the S5 Anding Formation, the anomaly manifests as a broad, lump-like low-resistivity core that is extensively connected with the overlying low-resistivity background of the S4 Weathered Bedrock. This corresponds to a broad “water basin” formed at the S4/S5 interface under the influence of the lagged tensile deformation of the key stratum, where a large amount of high-potential energy water accumulates.

Middle Breakthrough Throat ($z= - 160{\mathrm{m}}$, Fig. 8c): Tracing downward to the bottom boundary of the S5 Anding Formation, the planar extent of the low-resistivity anomaly significantly contracts, yet the intensity remains high and vertically continuous. This indicates that the massive catchment basin successfully “penetrated” the dense Anding Formation through a relatively narrow vertical channel at this location.

Lower Discharge Column ($z= - 230{\mathrm{m}}$, Fig. 8d): Entering the middle of the S6 Zhiluo Formation, the low-resistivity anomaly further contracts and focuses into a concentrated column, reaching directly into the goaf.

This funnel-shaped structure of “shallow broad convergence — deep contracted conduction” accurately reconstructs the dynamic process of lagged water inrush: at the moment of the high-position key stratum breaking, the water accumulated in the upper broad space inrushes into the goaf at high speed along the concentrated fracture zone formed by shear failure.

2. Low-position water accumulation morphology in the initial section of the Goaf (${\mathrm{x}} \approx 0\sim 100{\mathrm{m}}$)

In the relative coordinate range of $0\sim 100{\mathrm{m}}$ (i.e., the goaf area lagging behind the current working face about $260\sim 360{\mathrm{m}}$), a significant low-resistivity anomaly zone is developed. As this region has entered the goaf compaction stage, its anomaly morphology exhibits typical characteristics of “settlement-induced water accumulation”:

Vertical development characteristics: The main body of this low-resistivity volume is located in the deep low-lying region below $z< - 120{\mathrm{m}}$ (corresponding to the bottom of the S5 Anding Formation and the caving zone), manifesting as water accumulation on the goaf floor. In contrast, above $- 120{\mathrm{m}}$, since the overlying strata (S4) have gradually re-compacted and closed, the fracture space has decreased. This results in a sharp contraction of the low-resistivity anomaly range, with only a weak upward extension observed in the local area of $60\sim 90{\mathrm{m}}.$

Lateral contraction characteristics: The anomaly body exhibits a distinct “O-ring” contraction effect in the dip direction (Y-axis). At the bottom, its dip distribution range is wide ($0\sim 360{\mathrm{m}}$), covering almost the entire width of the working face. However, as the horizon rises, influenced by the high degree of compaction in the center of the goaf, the low-resistivity anomaly gradually contracts towards one side of the dip ($150\sim 360{\mathrm{m}}$) and shows a tendency to tilt towards the strike direction.

Analysis suggests that this morphology represents the result of post-mining compaction and gravity differentiation, rather than an active water inrush channel. Since this area is located far behind the working face, the high-position fractures have largely closed (manifesting as high resistivity in the upper part). The residual water has migrated under gravity to the low-lying depressions at the bottom of the goaf or to the marginal zones where fracture connectivity is better, forming a relatively stable low-position water accumulation zone.

Comprehensive Analysis: The 3D reconstruction results in Fig. 8 reveal the complexity of fluid distribution within the goaf at Time T2: the region $0\sim 100{\mathrm{m}}$ reflects the characteristics of low-position water accumulation under post-mining compaction (closed top, water accumulation at bottom), whereas the region $250{\mathrm{m}}$ reflects the characteristics of water inrush breakthrough induced by lagged strata breakage (high-position development, vertical conduction). This spatial morphological differentiation provides strong support for the diagnostic conclusion of “lagged water inrush in the goaf.”

#### Spatiotemporal evolution characteristics of the water-conducting fracture zone with working face advancement

**Fig. 9:**
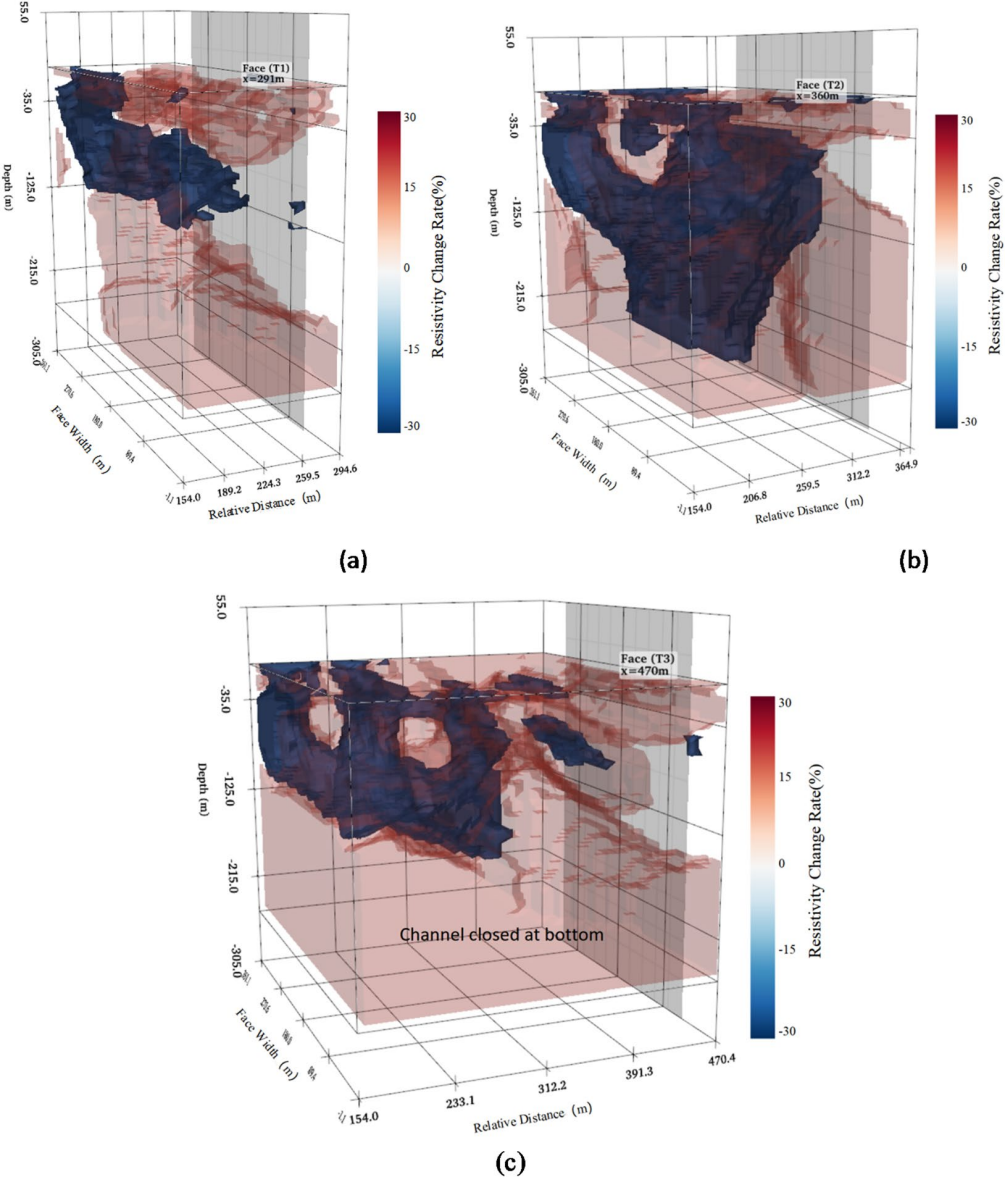
Three-dimensional spatiotemporal evolution characteristics of water-conducting fracture channels with working face advancement (The 3D visualization maps were generated using the **PyVista** library (https://docs.pyvista.org/) in **Python** (Version 3.9, https://www.python.org/).). (a) T1: High-position roof hanging and latent period (Lag distance < 110 m). (b) T2: Key stratum breaking and lagged water inrush outbreak (Lag distance ≈ 110 m). (c) T3: Bottom compaction and channel self-healing (Lag distance ≈ 220 m).

The continuous advancement of the working face induces dynamic failure and stress redistribution in the overlying strata. Selecting the center of the lagged water inrush anomaly (${\mathrm{x}}=250{\mathrm{m}}$) as the core observation object, and combining the electrical evolution characteristics from Stages T1 to T3, this section analyzes the evolution law of the water inrush channel within the goaf: “high-position hanging roof — lagged breakthrough — bottom closure,” as shown in Fig. 9.

1. Early post-mining stage and high-position roof suspension stage (T1, Sep. 4): The “Latent Period” of Hazard

At this time, the working face had advanced to $x=291{\mathrm{m}}$. In the deeper (older) goaf ($150\sim 200{\mathrm{m}}$), Fig. 9(a) shows scattered low-resistivity anomalies in the shallow region ($- 80\sim 0{\mathrm{m}}$). This corresponds to the retention of initial infiltration water from the S1 horizon in local bed separation fractures. However, the bottom of this region exhibits large-scale red high resistivity, indicating that no penetrating water-conducting channel has formed. At the observation point ($x=250{\mathrm{m}}$), the area generally manifests as a red high-resistivity background, with no obvious low-resistivity anomaly development.

The analysis suggests that at this time, the area at ${\mathrm{x}}=250{\mathrm{m}}$ is in the early post-mining stage. Although the low-level immediate roof has caved (manifesting as air-filled high resistivity), the upper S4 Weathered Bedrock and Key Strata remain in a state of a “suspended plate” and have not yet broken, due to the relatively small suspended span. Consequently, the water inrush hazard is in a latent period.

2. Key Stratum Breaking and Lagged Water Inrush Stage (T2, Sep. 12): The “Outbreak Period” of Hazard

At this time, the working face had advanced to $x=360{\mathrm{m}}$, and the distance to the water inrush center ($x=250{\mathrm{m}}$) was approximately$110{\mathrm{m}}$.

In Fig. 9(b), apart from the persistence of scattered anomalies at $150\sim 200{\mathrm{m}}$, the most significant change occurred in the central region at $x=250{\mathrm{m}}$. A typical funnel-shaped low-resistivity anomaly instantly appeared within the originally high-resistivity background. This anomaly body is wide at the top, contracts downward, and penetrates through the strata, constituting a vertical channel connecting the upper aquifer and the lower goaf.

Analysis suggests that when the lag distance reaches approximately $110{\mathrm{m}}$, the high-position key stratum reaches its limit breaking step (or interval) and undergoes lagged breaking. The roof water from the S4 formation inrushed instantly along the opened fractures, forming the funnel-shaped water-conducting channel seen in the figure, marking the occurrence of the lagged water inrush disaster.

3. Bottom compaction and channel blockage stage (T3, Sep. 25): The “Attenuation Period” of Hazard

At this time, the working face had advanced to $x=470{\mathrm{m}}$, and the water inrush center ($x=250{\mathrm{m}}$) was deeply buried in the compacted zone $220{\mathrm{m}}$ behind the working face.

In Fig. 9(c), scattered low-resistivity anomalies at $x=150\sim 200m$ and other areas still partially exist, reflecting the stability of local water accumulation in the goaf. The most critical change lies at $x=250{\mathrm{m}}$. The bottom of the funnel-shaped anomaly observed in Stage T2 (below − 120 m has completely disappeared and transformed into red high resistivity. The low-resistivity anomalies mainly remain in the interlayer region from − 120 m to -80 m and parts of the region from − 80 m to 0.

Analysis suggests that this phenomenon of “bottom disappearance and upper-middle retention” reveals the self-healing mechanism of the goaf overburden. With the compaction under the overlying load of the goaf, fractures in the caving zone at the bottom close first, or accumulated water is discharged, causing the water-conducting channel to be severed at the bottom (resulting in increased resistivity). However, in the high-position region from − 120 m to -80 m (corresponding to the middle-upper part of the fracture zone), due to the relatively lower degree of compaction, some bed separation fractures have not yet fully closed. This results in the retention of some residual water bodies here, but the hydraulic connection for downward recharge to the goaf has been lost. The spatiotemporal evolution characteristics in Fig. 9 comprehensively record the entire process of “bottom compaction cutoff” of the water-conducting channel. The disappearance of the bottom of the funnel-shaped anomaly in Stage T3 provides strong evidence that, as the working face advances beyond a certain distance (approx. 220 m), the compaction effect at the goaf bottom can effectively block the lagged water inrush channel. This transforms the water hazard from a “through-going type” to a “high-position retention type,” significantly reducing the risk of water inrush.

### Geological-geophysical response mechanism of lagged water inrush incubation and channel evolution in Goaf

Based on the characteristics of “spatial lag” and “funnel-shaped channels” revealed by the 3D electrical imaging in the previous text, this section introduces Key Stratum Theory and Rock Mass Structural Mechanics. It elucidates the disaster-causing mechanism behind the geophysical anomalies from the perspectives of stress evolution and medium properties.

#### Hysteresis effect model of “Limit Span” breaking of high-position key strata

The lagged water inrush phenomenon at $110{\mathrm{m}}$ confirmed by monitoring is essentially a dynamic response of the overburden transforming from “elastic plate-beam suspension” to “plastic breaking instability.”

1. Shielding Mechanism of the “Plate-Beam Effect”. Unlike the low-level immediate roof which exhibits “caving immediately upon mining,” the upper hard rock layer (High-position Key Stratum) of the S5 Anding Formation possesses significant bending stiffness. In the early post-mining stage (lag distance $<110{\mathrm{m}}$), the key stratum forms a suspended structure similar to a “plate-beam.” This structure utilizes its own tensile strength to bear the load of the overlying strata, temporarily blocking the upward development of water-conducting fractures. This mechanical shielding effect explains why, in Stage T1, the electrical structure of the S5 aquifuge remained intact despite mining disturbances in the deep sections.
2. Mutation Mechanism of “Limit Span”. Rock mechanics theory indicates that suspended strata possess a “limit breaking step” (or interval). When the working face advances to a lag of approximately $110{\mathrm{m}}$ (or even less), the span of the goaf reaches this threshold, and the key stratum undergoes instantaneous instability and fracturing. The accumulated elastic potential energy is released suddenly, causing penetrating shear failure in the S5 aquifuge. Therefore, the “mutation” observed in resistivity imaging is a direct mapping in the geophysical field of the stress field mutation caused by the breaking of the key stratum.

It should be noted that the specific lag distance of 110 m is a case-specific value for the 122105 working face. According to the key stratum theory, the initial breaking span (*L*) of a hard rock layer can be estimated by the fixed-beam model: $L=h\sqrt {2{\sigma _t}/q}$, where *h* is the thickness of the key stratum, ${\sigma _t}$ is the tensile strength, and *q* is the uniform load acting on the stratum. While this numerical value may vary across different mines in Northern Shaanxi due to variations in *h* and *q*, the underlying mechanical mechanism—lagged water inrush triggered by the periodic weighting of high-position key strata—represents a general evolutionary law for ultra-thick coal seam mining. Furthermore, the lag distance is sensitive to mining intensity; an increase in the advance rate (advance speed) typically leads to a larger spatial lag distance because the fracture development in the overburden is a time-dependent process involving rock creep and cumulative damage.

To quantitatively verify this, the mechanical parameters of the high-position key stratum (Anding Formation) from the mine’s geological report were utilized. With an average thickness (*h*) of $45{\mathrm{m}}$, a tensile strength (${\sigma _t}$) of $2.4\,\,{\mathrm{MPa}}$, and a calculated uniform load (*q*) of $0.8\,\,{\mathrm{MPa}}$, the theoretical initial breaking span is calculated as:

$$L=45 \times \sqrt {\frac{{2 \times 2.4}}{{0.8}}} \approx 110.2{\mathrm{m}}$$

This theoretical value ($110.2{\mathrm{m}}$) is in excellent agreement with the monitored lag distance of approximately $110{\mathrm{m}}$ (Fig. 7c), providing robust quantitative evidence that the lagged water inrush is indeed triggered by the first breakage of the high-position key stratum.

#### Fluid-solid coupling disaster mechanism of “bed separation tensile — shear conduction”

The “large top, small bottom” funnel-shaped anomaly revealed in Fig. 8 is, in physical essence, the product of fluid-solid coupling between mining-induced fractures of different properties and water migration.

First, the “broad funnel” at the top represents the negative pressure accumulation mechanism of bed separation spaces. In the upper section (corresponding to the S3/S4 interface), influenced by the bending subsidence prior to the breaking of the key stratum, “tensile failure” occurs first at the location with the weakest interlayer bonding, forming horizontal bed separation fractures. This volume expansion effect generates local negative pressure, inducing the water from roof aquifers to converge toward the center of the bed separation. Therefore, the “broad low-resistivity top” on the electrical map corresponds mechanically to a “horizontal bed separation catchment basin.”

Second, the “contracted neck” at the bottom represents the gravity conduction mechanism of broken fractures. In the lower section (corresponding to S5/S6 horizons), with the final rupture of the key stratum, the rock mass undergoes “shear failure,” forming a zone of near-vertical broken fractures. This region not only experiences a surge in permeability but also connects with the negative pressure zone of the goaf. The water accumulated in the upper bed separations discharges downward at high speed along these vertical fractures driven by gravity. Therefore, the “contracted conducting pipe” on the time-lapse resistivity map corresponds mechanically to a “vertical shear water-conducting channel.”

#### Self-healing mechanism of channels based on the theory of “differential compaction”

The phenomenon of “bottom high-resistivity closure and upper low-resistivity residue” monitored in Stage T3 reveals the differential compaction characteristics of the goaf overburden in the vertical direction.

1. “Granular Compaction” Characteristics of the Caving Zone The bottom of the goaf (the bottom of S6 and the coal seam space) belongs to the “caved zone of loose material.” The rock in this region is broken and exhibits a large bulking factor. With the re-application of the overlying load, the loose rock blocks are highly prone to rotation and crushing, causing pores to be rapidly compacted and filled. This “high compressibility” causes the water-conducting channels at the bottom to close first (effectively closing the “physical valve”) and cuts off the water recharge. Consequently, this manifests as a rapid rebound of resistivity at the bottom on the electrical map.
2. “Voussoir Beam” Support Characteristics of the Fracture Zone In contrast, the middle-upper strata (S5 horizon) are located in the “regular breaking zone.” The broken rock blocks form a “Voussoir (Masonry) beam” articulated structure. This structure possesses a certain bearing capacity, enabling it to resist part of the overlying pressure and delay fracture closure. Therefore, even though the bottom has been compacted, the middle-upper parts still retain some fracture space for the retention of residual water (or “suspended water”), leading to a slower dissipation of the low-resistivity anomaly in the middle part of the time-lapse resistivity map.

Furthermore, to quantitatively evaluate the fluid-solid coupling and the “self-healing” degree of the water-conducting channels, we introduce the Resistivity Recovery Index ($\Delta \eta$) as a proxy indicator for fracture closure. Due to the spatial resolution limitations of surface-based ERT at a depth of 250 m, directly measuring millimetric fracture apertures is highly challenging. However, the electrical response serves as a sensitive proxy. Monitoring data indicates that in the compaction zone, the resistivity change rate ($\Delta \rho$) recovered from an extreme low of -30% (during the T2 water inrush outbreak) to -5% (at stage T3). This represents a remarkable 83.3% quantitative recovery toward the initial background geoelectric state ($\Delta \eta = 83.3\%$). This recovery threshold numerically signifies the structural re-compaction of the caving materials and the effective closure of the macroscopic water-conducting pathways.

## Comprehensive verification of monitoring results

**Fig. 10:**
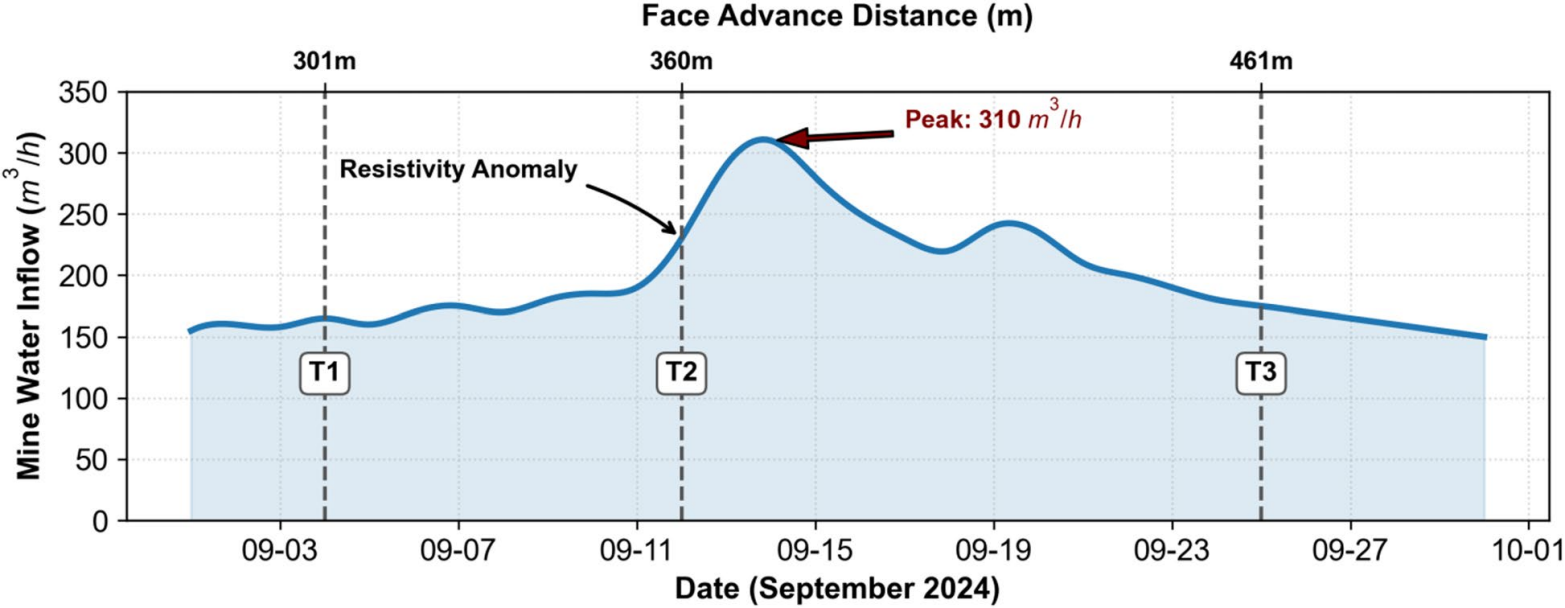
Spatiotemporal correspondence between mine water inflow and mining advance.

To confirm that the “funnel-shaped” low-resistivity anomalies captured in the resistivity inversion are indeed water-conducting channels, this section introduces actual measured hydrological dynamic data from the mine (Fig. 10). Cross-verification of multi-dimensional data is conducted from two dimensions: “temporal synchronization” and “spatial correspondence.” Based on this, targeted water hazard prevention and control strategies are proposed.

### Multi-dimensional coupling verification of “time-space-quantity” based on mine water inflow dynamics

By comparing and analyzing the time-lapse resistivity monitoring results with the synchronous mine total water inflow monitoring curve, the two exhibit a high degree of “consistency between flow field and geoelectric field response” in terms of spatiotemporal evolution.

1. Verification of strong temporal synchronization of “Anomaly Mutation — water volume surge”

As shown in Fig. 10, the geoelectric field response and hydrological dynamics present a clear causal chain.

At Time T1 (Sep. 13): The mine water inflow was stable in the range of $160\sim 170{{\mathrm{m}}^3}/{\mathrm{h}}$. At this time, resistivity imaging showed that the overburden was dominated by high-resistivity bed separation fractures, indicating that although mining-induced fractures had developed, an effective channel penetrating the S5 aquifuge had not yet formed, and the hydraulic connection had not been established.

At Time T2 (Sep. 12): As indicated previously, a “funnel-shaped” strong low-resistivity anomaly penetrating the S4-S6 horizons instantly appeared in the resistivity profile, marking the breakthrough of the aquifuge. The mine water inflow curve ended its steady state and showed a significant “upward trend” (jumping to $\:230{\mathrm{m}}^{3}/\mathrm{h}$). This synchronization confirms the immediate capture capability of resistivity anomalies for the formation of water inrush channels.

Subsequently on Sep. 13–14: Within 24–48 h after the T2 channel breakthrough, the water accumulated at high positions inrushed at high speed through the fractures, causing the water inflow to climb rapidly to a peak of $\:310{\mathrm{m}}^{3}/\mathrm{h}$. This short time lag characteristic of “peaking the day after T2 breakthrough” conforms to the dynamic law of the instantaneous release of high-position bed separation water.

At Time T3 (Sep. 25): As the resistivity imaging showed the closure of the channel bottom (compaction effect), the water inflow also dropped significantly to around $175{{\mathrm{m}}^3}/{\mathrm{h}}$, tending towards a new dynamic equilibrium.

2. Spatial correspondence verification of “Lag Distance — Working Face Position”

Based on the spatial back-analysis using the top X-axis (mining footage) of Fig. 10, the geometric location of the water inrush can be pinpointed. Time T2 (Sep. 12) corresponds to the starting point of the sudden change in water inflow. At this moment, the top X-axis shows that the working face had advanced to the position of ${\mathrm{x}}=360{\mathrm{m}}$, while the center of the water inrush channel (the funnel neck) accurately identified by time-lapse resistivity imaging is located at the relative monitoring coordinate of $250\,\,{\mathrm{m}}.$

Based on this, the lag distance can be calculated as $110\,\,{\mathrm{m}}$. This result rules out the possibility of water inrush caused by geological structures (as structural positions are fixed and do not move with the working face). It confirms that this water inrush is a dynamic disaster induced by the lagged breaking of the high-position key stratum after the goaf range reached the “limit breaking step (approx. $110\,\,{\mathrm{m}}$).”

The high degree of spatial-temporal convergence between the ERT-visualized channel and the mine water inflow surge (Time T2) provides the strongest evidence for the physical reality of the deep anomalies. Such a high-fidelity correlation rules out the possibility of the 250 m funnel being a random inversion artifact, as computational ghosts would not systematically synchronize with the dynamic advancement of the working face and the independent hydrological response.

### Stereoscopic prevention and control strategy for roof water hazards based on the law of “spatiotemporal lag”

In view of the critical law revealed by this study that the “center of water inrush lags behind the working face by $110\,\,{\mathrm{m}}$" in fully mechanized top-coal caving of ultra-thick coal seams, the traditional prevention mode of “focusing only on pre-mining exploration while ignoring post-mining monitoring” contains significant blind spots. Therefore, the following stereoscopic prevention and control strategies are proposed:

1. “Backward Shifting” Strategy of Monitoring Field of View. Changing the previous single-minded approach of focusing only on pre-mining exploration ahead of the working face, a “full spatiotemporal” monitoring system should be constructed.

Delineation of Lagged Risk Zones: The goaf area $0\sim 150{\mathrm{m}}$ behind the working face is designated as the “high-risk zone for roof water hazards.”

Deployment of Dynamic Monitoring: Within this range, ground time-lapse electrical monitoring should be continuously conducted to focus on capturing precursor information of high-position strata mutating from “high resistivity” to “low resistivity,” thereby eliminating post-mining blind spots.

2. “Zoned and Layered” Precision Control Strategy for Goaf. Based on the characteristics of “funnel-shaped” channels and “low-position water accumulation” revealed by resistivity imaging, differential control should be implemented:

Implementation of “High-Position Interception” for the Water Inrush Sensitive Zone (Funnel Zone) near $110{\mathrm{m}}$ Lag: Before mining, high-position roof directional long boreholes should be utilized to pre-drain the S4 Weathered Bedrock water. This reduces the potential energy of the water level in the “catchment basin” at the top of the “funnel” in advance, cutting off the water inrush source, and realizing the transformation from “passive water resistance” to “active water control.”

Implementation of “Low-Position Drainage” for the Compacted Zone (Water Accumulation Zone) with Lag $0\sim 100{\mathrm{m}}$: Buried pipe extraction in the goaf or floor low-position borehole drainage should be strengthened. This prevents residual accumulated water from converging in low-lying areas of the floor and softening the rock strata, ensuring that goaf water does not flow back towards the working face.

## Conclusions

In response to the engineering challenges of high concealment and lagged water inrush locations associated with roof water hazards during fully mechanized top-coal caving of ultra-thick coal seams in the aeolian sand area of Northern Shaanxi, this paper conducted long-distance dynamic monitoring using the “deep-buried electrode” ground pseudo-3D time-lapse resistivity method. Combined with mine hydrological observation data and key stratum breaking theory, the study revealed the incubation mechanism and evolution laws of lagged water inrush in the goaf. The main conclusions are as follows:

1. Overcame the technical bottleneck of deep detection under high-resistivity shielding in aeolian sand areas. Addressing the problem of “high-resistivity shielding” formed by dry surface aeolian sand, a deep-buried electrode technique using “manual excavation + wet soil coupling” was proposed, effectively reducing grounding resistance. A data processing workflow of “pseudo-3D acquisition + anisotropy-constrained inversion + time-lapse ratio imaging” was constructed. This successfully extracted weak mining-induced electrical response signals from depths below 250 m and realized the dynamic visualization of the entire process of mining-induced fracture development, providing a technical paradigm for deep detection under similar geological conditions.
2. Revealed the spatiotemporal evolution laws and mechanical mechanisms of “lagged water inrush” in the goaf. The monitoring results indicate that the water inrush center caused by high-intensity mining of ultra-thick coal seams is not located at the advancing line of the working face but exhibits significant “spatiotemporal lag” characteristics. The water inrush center in this study lagged behind the working face by approximately 110 m, a distance that coincides highly with the “limit breaking step” of the high-position hard key stratum. This indicates that the “plate-beam” suspension structure of the high-position key stratum shielded the water-conducting channels in the early post-mining stage, and the “brittle fracture” that occurred when the goaf span reached the limit threshold was the fundamental mechanical cause inducing the lagged water inrush.
3. Constructed a fluid-solid coupling model of the “large top, small bottom” funnel-shaped water inrush channel. The 3D resistivity imaging accurately delineated the stereoscopic morphology of the lagged water inrush channel: the upper part (S3/S4 interface) presents a broad lump-like low-resistivity zone, corresponding to the “horizontal bed separation catchment basin” induced by the bending and subsidence of the key stratum; the lower part (S5/S6 horizons) presents a contracted columnar low-resistivity zone, corresponding to the “vertical shear conducting pipe” formed by the breaking of the key stratum. The essence of the water inrush is a fluid-solid coupling dynamic process in which the water accumulated in the upper bed separation inrushes into the goaf at high speed along the vertical fractures after instantaneously losing floor support.
4. Verified the “consistency between flow field and geoelectric field response” of resistivity imaging and mine water inflow.Comparative analysis of multi-source data confirmed that the Time T2 (Sep. 12, the starting point of channel breakthrough) captured by the ground electrical method was completely synchronous with the moment of the “mutation rise” in the mine water inflow curve. Furthermore, the water inflow reached its peak (310 m³/h) within 24 to 48 h thereafter. This high degree of “temporal synchronization” confirms that the time-lapse high-density electrical method possesses the capability for immediate capture and early warning of concealed water-conducting channels in the goaf.
5. Elucidated the “self-healing” mechanism of water-conducting channels and proposed stereoscopic prevention and control strategies. Long-term monitoring showed that as the working face advanced to a greater distance (lag > 150 m), influenced by the granular compaction effect of the caving zone in the goaf, the funnel-shaped channel closed first at the bottom (resistivity rebound), leaving only some suspended water in the middle and high positions. Based on this, it is suggested to transform the traditional model and designate the area 0 ~ 150 m behind the working face as a key prevention and control zone. A zoned governance strategy of “high-position interception (targeting the funnel zone lagging 110 m)” and “low-position drainage (targeting the compacted zone lagging < 100 m)” should be implemented.

## Data Availability

The data that support the findings of this study are available from the corresponding author upon reasonable request.
